# Comparison of clinical and survival characteristics between prostate cancer patients of PSA-based screening and clinical diagnosis in China

**DOI:** 10.18632/oncotarget.20787

**Published:** 2017-09-08

**Authors:** Libo Xu, Jinguo Wang, Baofeng Guo, Haixia Zhang, Kaichen Wang, Ding Wang, Chang Dai, Ling Zhang, Xuejian Zhao

**Affiliations:** ^1^ Department of Pathophysiology, College of Basic Medical Science, Jilin University, Changchun, China; ^2^ Department of Urology, The First Hospital of Jilin University, Changchun, China; ^3^ Department of Surgery, China-Japan Union Hospital of Jilin University, Changchun, China; ^4^ Beijing Chaoyang District Center for Disease Control and Prevention, Beijing, China; ^5^ Department of Ophthalmology, Second Hospital of Jilin University, Changchun, China

**Keywords:** prostate cancer, PSA-based mass screening, prostate-cancer specific mortality, overall survival, metastases

## Abstract

Prostate-specific antigen (PSA)-based mass screening remains the most controversial topic in prostate cancer. PSA-based mass screening has not been widely used in China yet. The aim of our study was to evaluate the effect of the PSA-based screening in China. The cohort consisted of 1,012 prostate cancer patients. Data were retrospectively collected and clinical characteristics of the cohorts were investigated. Survival was analyzed for prostatic carcinoma of both PSA screened and clinically diagnosed patients according to clinical characteristics and the National Comprehensive Cancer Network (NCCN) risk classification. Cox Proportional Hazards Model analysis was done for risk predictor identification. The median age was 71 years old. Five-year overall and prostate-cancer-specific survival in prostatic adenocarcinoma patients were 77.52% and 79.65%; 10-year survivals were 62.57% and 68.60%, respectively. Survival was significantly poorer in patients with metastases and non-curative management. T staging and Gleason score by NCCN classification effectively stratified prostatic adenocarcinoma patients into different risk groups. T staging was a significant predictor of survival by COX Proportional Hazard Model. PSA screened patients had a significantly higher percentage diagnosed in early stage. PSA screened prostatic adenocarcinoma patients had a better prognosis in both overall and prostate cancer-specific survivals. This Chinese cohort had a lower overall and prostate cancer survival rate than it is reported in western countries. The incidence of early-stage prostate cancer found in PSA-based mass screening was high and there were significant differences in both overall and prostate cancer-specific survival between the PSA-screened and clinically diagnosed patients.

## INTRODUCTION

Prostate cancer is one of the most common malignancies among males, especially in developed countries [1]. It is estimated that over 160,000 new cases of prostate cancer will be diagnosed in 2017 in the United States, accounting for 19% of all cancer cases [2]. In European countries too, prostate cancer is the most common non-skin cancer in men over 70 years old [3]. With the development of novel diagnostic and therapeutic technologies in the past decades, a drop of more than 25% in cancer death rates was witnessed since early 1990s in the United States [4]. Reduction in prostate cancer mortality during last decades was also reported in European counties with long term follow-up [5].

By comparison, Asian countries including China were reported as having a relatively low rate [4]. However a sharp increase in prostate cancer incidence in China was recorded in the past few decades, probably due to a combination of factors including ageing and dietary and lifestyle changes. Based on age-standardized rate per 100,000 analysis, an epidemiological study of Cancer Institute of Shanghai reported an incidence increase from 0.48 in 1960s to 2.41 in 1990s. In recent years another worldwide study showed an elevated incidence rate in China from 4.48 to 13.33 and an over 5-fold mortality increase as well between 2009 and 2013 [1, 5].

Incidence rates from 1975 through 2013 in the United States showed a dramatic increase during late 1980s and early 1990s and then a marked drop from 1992 to 1995. Thereafter the incidence declined annually and showed a sharp reduction of more than 10% in particular from 2010 to 2013 [6, 7]. The dramatic shifts were considered a reflection of changing patterns of prostate-specific antigen (PSA) mass screening, which was recommended initially by the American Cancer Society (ACS) and the American Urological Association (AUA) from the late 1980s [2, 7]. However, the 2008 and 2012 US Preventive Services Task Force (USPSTF) guidelines discouraged routine PSA screening because of concerns about the uncertain balance between the benefits of screening for early detection and the harmful effects of overdiagnosis and the resultant overtreatment [8, 9]. PSA-based mass screening is therefore one of the most controversial topics in prostate cancer diagnosis.

Because of the geographical differences in incidence of prostate cancer, there has been less epidemiological research in Asian countries. PSA-based mass screening has not been widely used in China yet. The question whether PSA-based mass screening can reduce mortality in Chinese prostate cancer patients and how to balance the uncertain benefits and risks of screening remains to be determined. Hence, to evaluate the effect of PSA-based mass screening in China, as early as 1996, the Center of Diagnosis, Treatment and Research of Prostate Disease of Jilin University launched a PSA-based mass screening program for prostate cancer in a Chinese cohort in Changchun, China, in cooperation with Japan International Cooperation Agency (JICA).

The present study investigated the clinical characteristics and survival of Chinese patients to uncover the epidemiological features of prostate cancer in China with the hope of identifying reliable diagnostic and prognostic risk factors. It also compares prostate cancer between patients detected by PSA-based mass screening and those identified by clinical diagnosis, to evaluate the effect of PSA-based mass screening. We hoped to aid clinicians on better selection in management options to enhance treatment efficacy as well as guide health authorities for policy-making on prostate cancer in China.

## RESULTS

Of the 1,012 patients in the study, 984 men were diagnosed as having prostatic adenocarcinoma and 28 as having other types of prostate cancer, including squamous cell carcinoma, prostatic sarcomas and transitional cell carcinomas. The median age of the whole cohort was 71 years.

### Clinical characteristics of the whole cohort

The distribution of clinical characteristics was analyzed and is shown in Table 1 based on median age. The median PSA level was significant higher in the group of median Age >71 years (median 25.25 and 29.3 ng/mL, respectively, *P*=0.0056). Also, there were significant differences in the year of diagnosis (*P*<0.0001), T staging (*P*=0.0002), management approach (*P*<0.0001) as well as follow-up time (*P*<0.0001).

**Table 1:** Patient clinical characteristics based on median age

Clinial characteristic	Whole cohort N>1012		Age<=71 N>529		Age>71 N>479		P value
**Median age, year**	71		65		76		
**Year of diagnosis**							*P*< 0.0001
1980-1990	68	6.7%	52	9.8%	16	3.3%	
1991-2000	268	26.6%	182	34.4%	86	18.0%	
2001-2012	672	66.7%	295	55.8%	377	78.7%	
**Clinical staging**							
T staging*							*P*= 0.0002
T1	55	5.5%	31	5.9%	24	5.0%	
T2	395	39.2%	192	36.3%	203	42.4%	
T3	120	11.9%	74	14.0%	46	9.6%	
T4	185	18.4%	124	23.4%	61	12.7%	
Missing data	253	25.1%	108	20.4%	145	30.3%	
**N staging**							N0 vs N1,2, *P*=0.1795
N0	531	52.7%	324	61.2%	207	43.2%	
N1,2	30	3.0%	22	4.2%	8	1.7%	
Nx	447	44.3%	183	34.6%	264	55.1%	
M staging*							M0 vs M1, *P*=0.4803
M0	518	51.4%	296	56.0%	222	46.3%	
M1	244	24.2%	133	25.1%	111	23.2%	
Mx	246	24.4%	100	18.9%	146	30.5%	
**Median PSA(IQR), ng/mL**	26.7	(0-1000)	25.25	(0-241.2)	29.3	(0.1-1000)	*P*=0.0056
Missing data	357	35.4%	239	45.2%	118	24.6%	
Gleason score*							*P*=0.1599
<=6	204	20.2%	104	19.7%	100	20.9%	
7	176	17.5%	77	14.6%	99	20.7%	
8	117	11.6%	46	8.7%	71	14.8%	
9	117	11.6%	62	11.7%	55	11.5%	
10	24	2.4%	12	2.3%	12	2.5%	
Missing data	370	36.7%	228	43.1%	142	29.6%	
**No. of all-cause death, no. (%)**	269	26.7%	144	27.2%	125	26.1%	
**No.of cancer specific death, no. (%)**	225	22.3%	127	24.0%	98	20.5%	
**Management approach**							*P*<0.0001
Curative	55	5.5%	46	8.7%	9	1.9%	
Non-curative	953	94.5%	483	91.3%	470	98.1%	
**Median follow-up time, year**	5	(0.08-23)	6	(0.08-23)	4	(0.1-23)	*P*<0.0001

IQR, interquartile range.

*Percentages may not add up to 100 due to rounding.

Given that most cancer deaths were attributable to the metastatic prostate cancer, the distribution of clinical characteristics based on metastatic status at diagnosis was further analyzed and is shown in Table 2. Men with metastases had a significantly higher median PSA level than those without metastases (70.05 ng/mL and 15.70 ng/mL, respectively, *P*<0.0001). Also, differences in T staging, Gleason score and follow-up time were significant between the two groups (*P*<0.0001).

**Table 2:** Patient clinical characteristics based on metastases status

Clinial characteristic	Whole N>763		Non-metastases N>519		Metastases N>244		P value
**Median age at diagnosis, year**	70		70		71		*P*=0.1998
Missing data	239						
**Year of diagnosis**							*P*=0.0008
1980-1990	68	8.9%	54	10.4%	14	5.7%	
1991-2000	265	34.7%	196	37.8%	69	28.3%	
2001-2012	430	56.4%	269	51.8%	161	66.0%	
**Clinical staging**							
**T staging**							*P*<0.0001
T1	55	7.2%	55	10.6%	0	0.0%	
T2	389	51.0%	261	50.3%	128	52.5%	
T3	116	15.2%	94	18.1%	22	9.0%	
T4	185	24.2%	91	17.5%	94	38.5%	
Missing data	18	2.4%	18	3.5%	0	0.0%	
**Median PSA(IQR), ng/mL**	25.4	(0-345)	15.70	(0-345)	70.05	(0-241.2)	*P*<0.0001
Missing data	320	41.9%	250	48.2%	70	28.7%	
**Gleason score**							*P*<0.0001
<=6	182	23.9%	146	28.1%	36	14.8%	
7	143	18.7%	92	17.7%	51	20.9%	
8	96	12.6%	48	9.2%	48	19.7%	
9	97	12.7%	54	10.4%	43	17.6%	
10	21	2.8%	16	3.1%	5	2.0%	
Missing data	224	29.4%	163	31.4%	61	25.0%	
**Management approach**							*P*=0.2452
Curative	49	6.4%	37	7.1%	12	4.9%	
Non-curative	714	93.6%	482	92.9%	232	95.1%	
**No.of all-cause death, no.(%)**	264	34.6%	166	32.0%	98	40.2%	
**No.of prostate cancer specific death, no.(%)**	221	29.0%	128	24.7%	93	38.1%	
**Median follow-up time, year**	6	(0.08-23)	6	(0.08-23)	5	(0.41-16)	*P*<0.0001

IQR, interquartile range.

*Percentages may not add up to 100 due to rounding.

### PSA-based mass screening and clinically diagnosed prostate cancer patients

#### Overall survival and prostate cancer-specific mortality

Survival is one of the most important indicators for PSA-based mass screening evaluation. Hence, the overall and prostate cancer-specific survival in both PSA-based mass screening and clinically diagnosed prostate cancer patients as a whole was analyzed firstly in this study. To reduce the heterogeneity, prostate cancer in special types were excluded and therefore, 984 men with prostatic adenocarcinoma were included for survival analysis.

(i) Basic characteristics and survival

**Figure 1:**
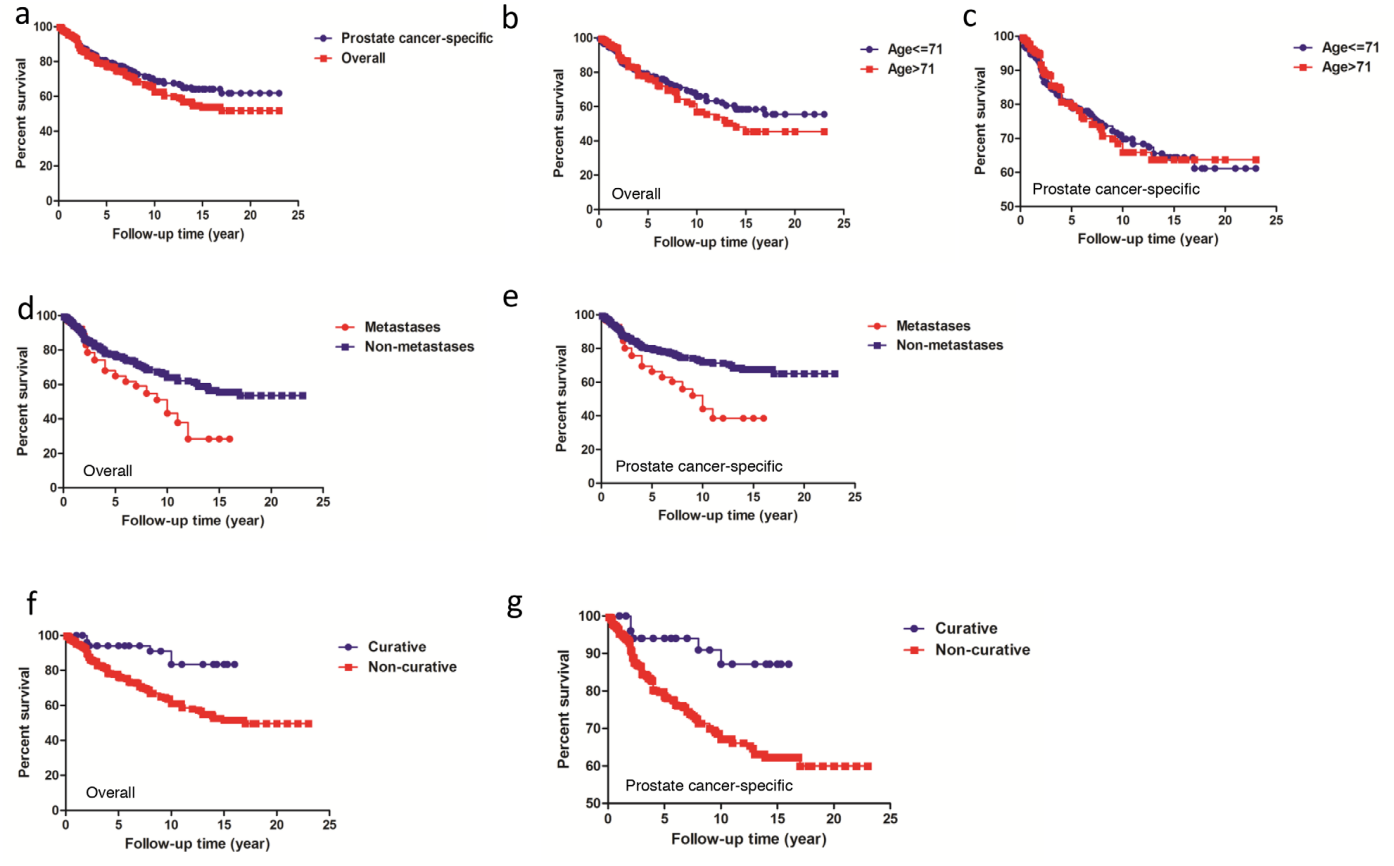
Overall and prostate cancer-specific survivals based on median age, metastatic status and management approach **(a)** Comparison of overall and prostate cancer-specific survival for the whole cohort, *P*=0.0409. **(b**, **c)** Overall and prostate cancer-specific survival comparison for men between age 71 or less and age>71. **(d**, **e)** Overall and prostate cancer-specific survival comparision for men with/without metastases. **(f**, **g)** Overall and prostate cancer-specific survival comparision based on management approach.

Kaplan-Meier plots for overall and prostate cancer-specific survival of patients with prostatic adenocarcinoma are shown in Figure 1a. Five-year overall and prostate cancer-specific survivals were 77.52% and 79.65% respectively. The 10-year survivals were 62.57% and 68.60% respectively. Statistical significance was found between overall and prostate cancer-specific survivals (*P*=0.0409). Subsequently, the overall survival and prostate cancer-specific survival was compared between different category groups based on median age, metastatic status and management approach. There was no significant difference between patients based on median age (overall survival, Figure 1b, *P*=0.1691; prostate cancer-specific mortality, Figure 1c, *P*=0.8835). However, there were significant differences in survival regarding to metastatic status (overall survival, Figure 1d, *P*=0.0004; prostate cancer-specific mortality, Figure 1e, *P*<0.0001). Also curative management appeared to significantly improve the survival of patients in comparison to non-curative management (overall survival, Figure 1f, *P*=0.0010; prostate cancer-specific mortality, Figure 1g, *P*=0.0038).

(ii) NCCN classification and survival

The NCCN classification categorizes prostate cancer into low, intermediate and high risks based on PSA value, T stage and Gleason score. The intermediate risk group is defined as including at least one of the characteristics: PSA 10-20 ng/mL, American Joint Committee on Cancer tumor (T) category T2b-T2c or Gleason 7 [11]. Accordingly, we compared the overall survival and prostate cancer-specific mortality among different PSA, T staging and Gleason score subgroups individually and also among different NCCN risk categories.

**Figure 2:**
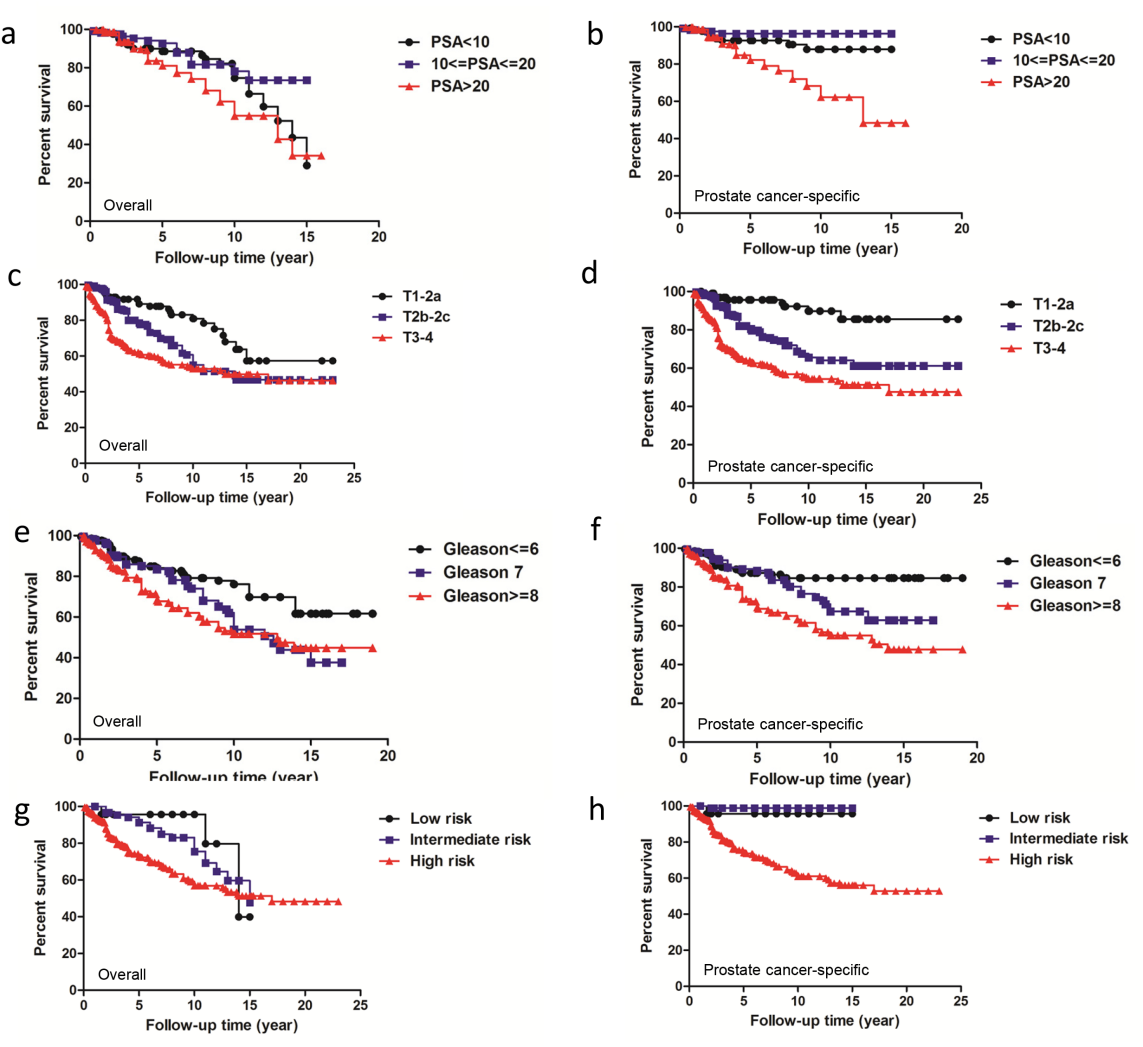
Overall survival and prostate cancer specific-survival according to each NCCN classification related factor PSA, T stage and Gleason score as well as NCCN risk categories **(a**, **b)** Overall and prostate cancer-specific survival comparison among patients with PSA<10, 10≤PSA≤20 and PSA>20. **(c**, **d)** Overall and prostate cancer-specific survival comparison among subcategories of patients with T1-T2a, T2b-T2c and T3-T4. **(e**, **f)** Overall and prostate cancer-specific survival comparison among patients with Gleason≤6, Gleason 7 and Gleason≥8. **(g**, **h)** Overall and prostate cancer-specific survival comparision based on the NCCN classification.

For survival comparison based on PSA levels, 639 patients with defined PSA level records were classified into three groups: PSA<10, 10≤PSA≤20 and PSA>20. Five-year overall survivals were 88.56%, 92.69% and 81.02% respectively and 10-year survivals were 74.65%, 78.14% and 54.93% respectively (Figure 2a, *P*=0.0044). Five-year prostate cancer-specific survivals were 92.59%, 96.36% and 82.16% respectively and 10-year survivals were 87.90%, 96.36% and 62.19% respectively (Figure 2b, *P*<0.0001).

T staging and Gleason score appeared to be better able to reduce the heterogeneity with regard to both overall survival and prostate cancer specific survivals. For T staging, 734 patients were subcategorized into three groups of T1-T2a, T2b-T2c and T3-T4. Five-year overall survivals were 89.10%, 78.53% and 61.19% respectively and 10-year survivals were 80.89%, 54.99% and 52.81% respectively (Figure 2c, *P*<0.0001). The five-year prostate cancer-specific survivals were 95.65%, 80.50% and 62.99%, respectively and 10-year survivals were 89.81%, 65.51% and 54.36% respectively (Figure 2d, *P*<0.0001). In 631 patients with Gleason≤6, Gleason 7 and Gleason≥8, five-year overall survivals were 84.15%, 83.49% and 68.32% respectively and 10-year survivals were 76.16%, 53.88% and 51.69% respectively (Figure 2e, *P*=0.0004). Five-year prostate cancer-specific survivals were 87.24%, 88.33% and 69.42%, respectively and 10-year survivals were 84.64%, 67.35% and 55.08%, respectively (Figure 2f, *P*<0.0001).

Subsequently, 803 patients were subcategorized as low, intermediate and high risk, respectively. As analyzed, five-year survivals were 95.65%, 91.29% and 72.81% respectively and 10-year survivals were 95.65%, 75.53% and 56.84% respectively (Figure 2g, *P*=0.0019). Five-year prostate cancer-specific survivals were 95.65%, 98.88% and 74.23% respectively and 10-year survivals were 95.65%, 98.88% and 60.93%, respectively (Figure 2h, *P*<0.0001).

(iii) Cox Proportional Hazards Model and survival

After a median follow-up of five years (IQR, 0.08-23 years) for the whole cohort, there were 269 deaths (26.58%) in total, 225 of which were prostate cancer-specific deaths (83.64%). Cox Proportional Harzard Model analysis for overall survival and prostate cancer-specific mortality of the whole cohort is shown in Table 3. Eight co-facters, including age at the time of diagnosis, year of diagnosis, PSA level, TNM staging, Gleason score and management approach, were analyzed with the model. T staging and year of diagnosis were significant predictors in overall survival (*P*=0.008 and *P*=0.030, respectively) whereas for prostate cancer-specific death, T staging was the only significant predictor of survival (*P*=0.044).

**Table 3:** Cox proportional hazard model for the whole cohort

Clinical characteristics	Overall survival			Prostate cancer-specific survival		
	HR	95% CI	*P*	HR	95% CI	*P*
Age at the time of diagnosis, year	1.016	0.98-1.053	0.401	0.999	0.951-1.05	0.976
Year of diagnosis	1.114	1.01-1.228	0.030	1.06	0.927-1.211	0.395
PSA, ng/mL	0.996	0.984-1.008	0.530	1.003	0.989-1.016	0.702
T staging	1.897	1.179-3.054	0.008	1.87	1.018-3.436	0.044
N staging	0.255	0.029-2.238	0.218	0.767	0.083-7.062	0.815
M staging	0.686	0.231-2.037	0.497	0.396	0.087-1.802	0.231
Gleason score	0.966	0.862-1.082	0.546	0.924	0.796-1.072	0.295
Management approach	0.509	0.173-1.501	0.221	1.096	0.296-4.064	0.891

95% CI, 95% confidence interval; HR, hazard ratio.

Cox Proportional Hazard Model analysis was performed for patients with prostatic adenocarcinoma (Table 4). Among 984 men with prostatic adenocarcinoma, there were 261 all-cause deaths in total and 217 deaths caused by prostate cancer (83.14%). Similarly, T staging and year of diagnosis were significant predictors of overall survival (*P*=0.006 and *P*=0.040, respectively). T staging was the only significant predictor of prostate-cancer-spcific survival (*P*=0.031).

**Table 4:** Cox proportional hazard model for prostatic adenocarcinoma patients

Clinical characteristics	Overall survival			Prostate cancer-specific survival		
	HR	95% CI	*P*	HR	95% CI	*P*
Age at the time of diagnosis, year	1.015	0.977-1.053	0.445	0.999	0.948-1.053	0.974
Year of diagnosis	1.11	1.005-1.226	0.040	1.047	0.913-1.201	0.51
PSA, ng/mL	0.996	0.984-1.009	0.564	1.004	0.99-1.018	0.571
T staging	2.027	1.226-3.349	0.006	2.068	1.067-4.01	0.031
N staging	0.221	0.025-1.956	0.175	0.67	0.07-6.441	0.729
M staging	0.649	0.214-1.968	0.445	0.35	0.071-1.719	0.196
Gleason score	1	0.883-1.133	0.998	0.987	0.838-1.162	0.872
Management approach	0.366	0.107-1.244	0.107	0.727	0.155-3.403	0.686

95% CI, 95% confidence interval; HR, hazard ratio.

### Clinical and survival characteristics in PSA-based mass screening and clinically diagnosed prostate cancer patients

Through the survival analysis, it was found that the overall and prostate cancer-specific survival was high in the Chinese cohort in comparison to it is in the western countries where PSA-based mass screening have been widely used for decades. In order to figure out whether PSA-based mass screening is contribute to mortality reduction in prostate cancer, we further compared the clinical and survival characteristics between PSA-based mass screening and clinically diagnosed prostate cancer patients.

Based on PSA-based mass screening, 383 men were diagnosed as having prostate cancer. Of these, 358 men had prostatic adenocarcinoma. 259 of them had biopsies and were diagnosed immediately and 99 men refused biopsies at first and were diagnosed years later when their PSA levels were very high. Therefore, in this study, to reduce discrepancies, these 99 men were subcategorized into another group as “PSA screened with later diagnosis”, whereas the other 259 patients were subcategorized as “PSA screened” patients. Clinical characteristics and survival were compared in three groups: 259 PSA screened patients, 99 PSA screened patients with later diagnosis, and 626 clinically diagnosed prostatic adenocarcinoma patients.

(i) Clinical characteristics

The clinical characteristics comparison is shown in Table 5. The distribution of TNM staging, Gleason score, median age, and follow-up time, as well as median PSA level were significantly different among PSA screened, PSA screened with later diagnosis, and the clinically diagnosed groups (*P*<0.0001). Also, there were significant differences in management approach (*P*=0.0049).

**Table 5:** Clinical characteristics based on PSA-based mass screening and clinical diagnosis

Clinical characteristics	Whole cohort N>984		PSA screened N>259		PSA screened with later diagnosis N>99		Clinically diagnosed N>617		P value
**Median age, year**	71		72		74		70		*P*<0.0001
**Clinical staging**									
T staging*									*P*<0.0001
T1	53	5.4%	18	6.9%	4	4.0%	31	5.0%	
T2	382	38.8%	229	88.4%	76	76.8%	77	12.3%	
T3	118	12.0%	6	2.3%	12	12.1%	100	16.0%	
T4	181	18.4%	5	1.9%	7	7.1%	169	27.0%	
Missing data	250	25.4%	1	0.4%	0	0.0%	249	39.8%	
N staging*									N0 vs N1,2, *P*=0.0458
N0	517	52.5%	140	54.1%	19	19.2%	358	57.2%	
N1,2	29	2.9%	2	0.8%	2	2.0%	25	4.0%	
Nx	438	44.5%	117	45.2%	78	78.8%	243	38.8%	
M staging*									M0 vs M1, *P*<0.0001
M0	500	50.8%	176	68.0%	32	32.3%	292	46.6%	
M1	242	24.6%	77	29.7%	65	65.7%	100	16.0%	
Mx	242	24.6%	6	2.3%	2	2.0%	234	37.4%	
**Median PSA(IQR), ng/mL**	28	(0-1000)	20	(0.22-124)	68	(5.1-150)	38	(0-1000)	*P*<0.0001
Missing data	347	35.3%	0	0.0%	0	0.0%	347	56.2%	
**Gleason score**									*P*<0.0001
<=6	206	20.9%	114	44.0%	22	22.2%	70	11.2%	
7	175	17.8%	85	32.8%	28	28.3%	62	9.9%	
8	115	11.7%	31	12.0%	30	30.3%	54	8.6%	
9	111	11.3%	26	10.0%	18	18.2%	67	10.7%	
10	24	2.4%	2	0.8%	1	1.0%	21	3.4%	
Missing data	353	35.9%	1	0.4%	0	0.0%	352	56.2%	
**Management approach**									*P*=0.0049
Curative	53	5.4%	24	9.3%	5	5.1%	24	3.8%	
Non-curative	931	94.6%	235	90.7%	94	94.9%	602	96.2%	
**No. of all-cause death, no. (%)**	261	26.5%	60	23.2%	40	40.4%	161	25.7%	
**No. of prostate cancer specific death, no. (%)**	217	22.1%	30	11.6%	37	37.4%	150	24.0%	
**Median follow-up time, year**	5	(0.08-23)	7	(1-16)	5	(1-11)	4	(0.08-23)	*P*<0.0001

IQR, interquartile range; *Percentages may not add up to 100 due to rounding.

To understand the detailed differences in distribution of clinical staging, Gleason score and management approach, we further compared the distribution within the subgroups of T1-2 versus T3-4; N0 versus N1-2, M0 versus M1, and curative versus non-curative management, as well as Gleason≤6, Gleason 7 and Gleason≥8.

**Figure 3:**
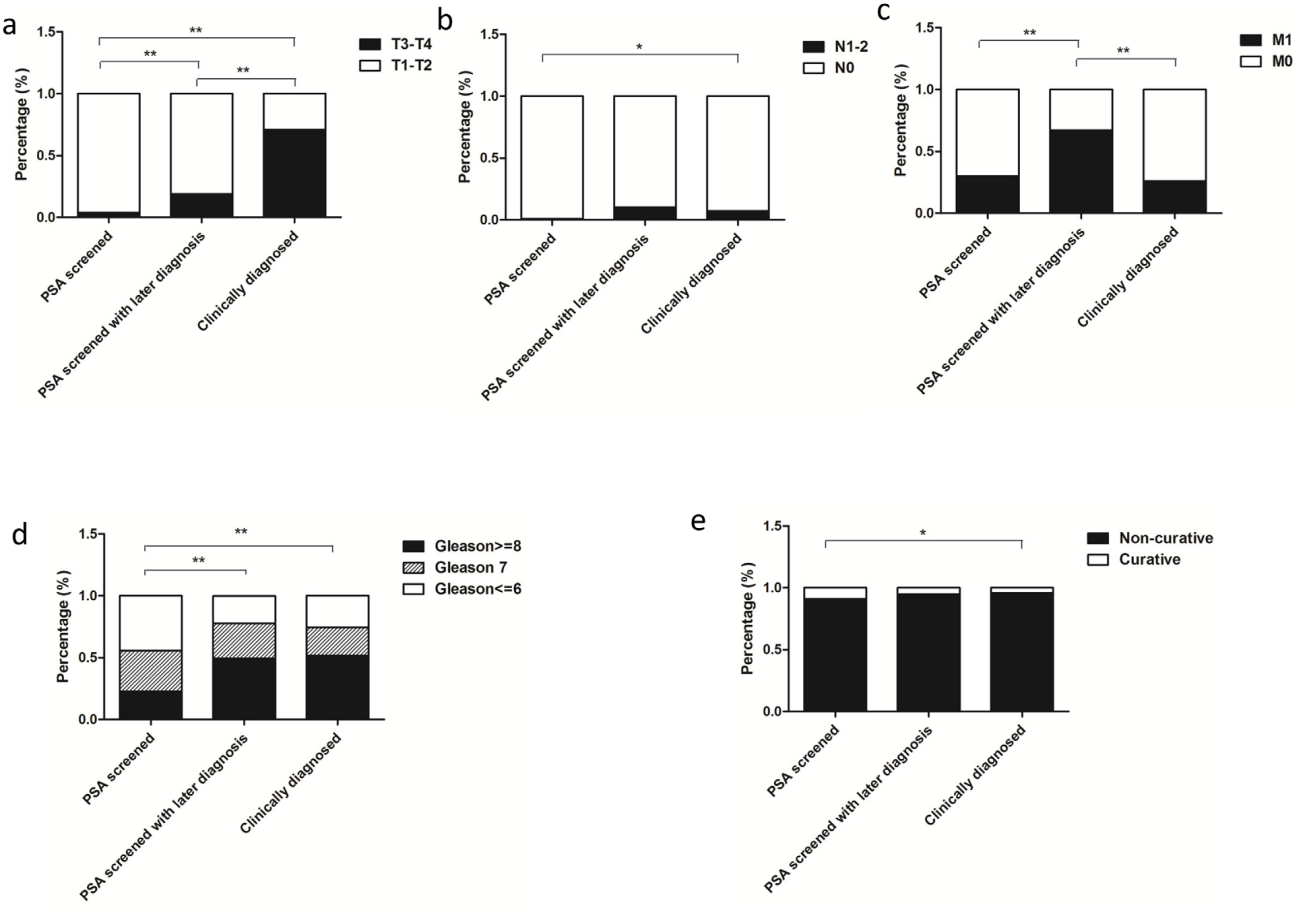
Clinical characteristics and survivals in PSA screened, PSA screened with later diagnosis and clinically diagnosed prostatic adenocarcinoma patients **(a)** T staging distribution. **(b)** Distribution of N staging. **(c)** Distribution of M staging. **(d)** Gleason score distribution. **(e)** Management approach comparison among three subcategories (*P*<0.0001). *P<0.05; **P<0.01.

For T staging, statistical significances were indicated among the subgroups (Figure 3a, *P*<0.0001) and PSA-screened patients had a higher percentage in T1-2 distribution (96%) in comparison to PSA-screened with later diagnosis (81%) and clinically diagnosed patients (29%). As to N staging, there was a significant difference between PSA screened and clinically diagnosed patients (Figure 3b, *P*=0.0146) but no significances were found in comparison between any other two groups (PSA-screened versus PSA screened with later diagnosis, *P*=0.0811; PSA-screened with later diagnosis versus clinically diagnosed, *P*=0.6425). On the contrary, there was no significance between PSA-screened and clinically diagnosed patients in M staging subgroups (Figure 3c, *P*=0.1763).

Gleason score comparison was indicated in Figure 3d. There were 114 (44.19%), 85 (32.95%) and 59 (22.87%) patients in PSA screened patients; 22 (22.22%), 28 (28.28%) and 49 (49.49%) in PSA screened with later diagnosis, and 70 (25.55%), 62 (22.63%) and 142 (51.82%) in clinically diagnosed group with defined status stratified in the subgroups of Gleason≤6, Gleason 7 and Gleason≥8, respectively. Significant differences were found in the groups of PSA-screened versus PSA-screened with later diagnosis and PSA-screened versus clinically diagnosed group (*P*<0.0001), whereas there was no significance in PSA-screened patients with later diagnosis compared with those diagnosed clinically (*P*=0.5048).

As to management approach (Figure 3e), a statistically significant difference was found between the PSA- screened and clinically diagnosed groups (*P*=0.0018), while no significant differences were found between any of other two groups (PSA-screened versus PSA-screened with later diagnosis, *P*=0.5786; PSA-screened with later diagnosis versus clinically diagnosed, *P*=0.2780).

(ii) Overall and prostate cancer-specific survivals

Since the purpose of PSA-based mass screening was to detect prostate cancer patients at an early stage in order to reduce mortality, survival was a very important factor in the evaluation of the effect of PSA mass screening. Therefore, Kaplan-Meier plots were compared among PSA-screened, PSA-screened with later diagnosis, and the clinically diagnosed groups in our study.

**Figure 4:**
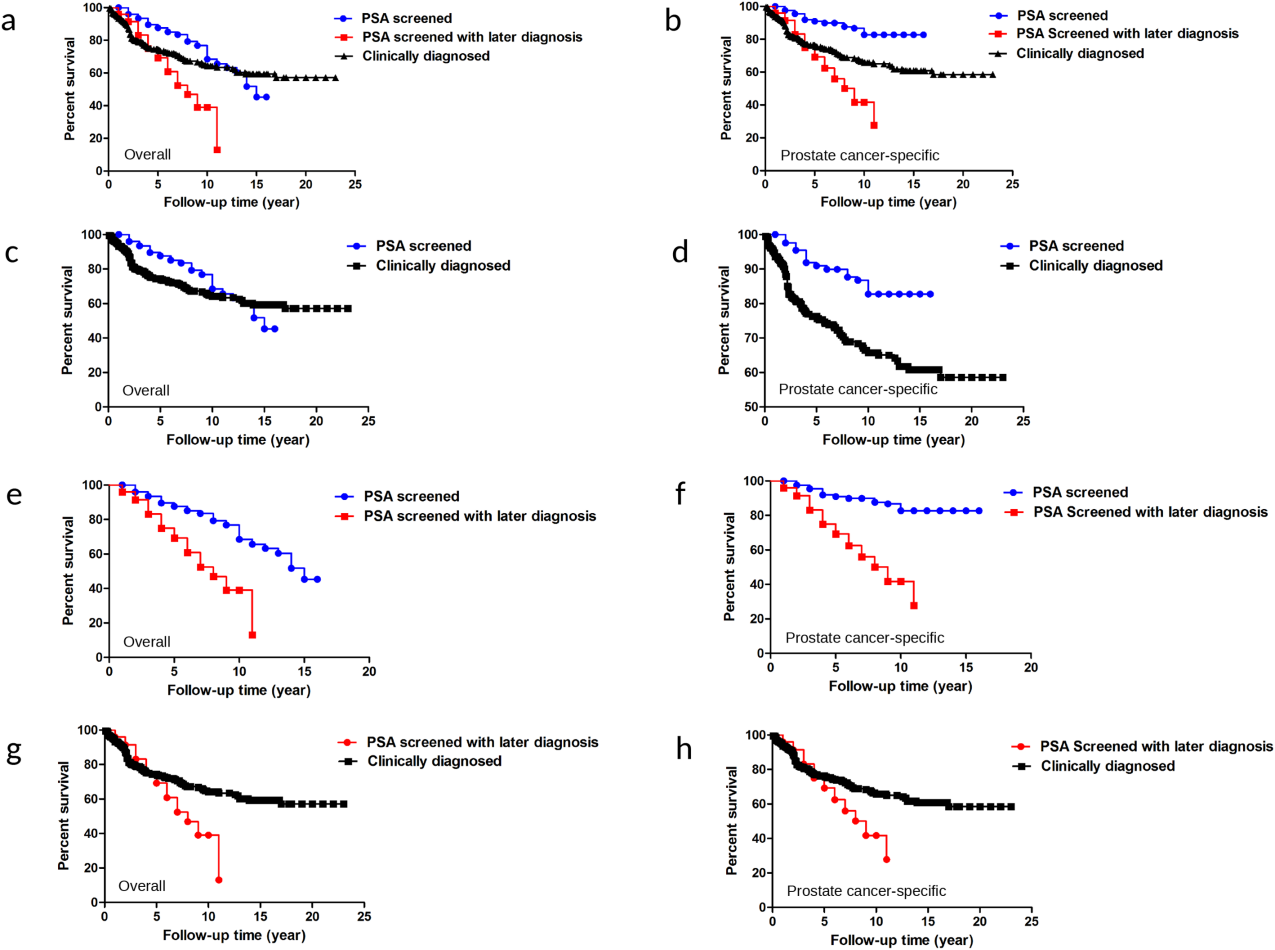
Overall and prostate cancer-specific survival comparison among PSA screened, PSA screened with later diagnosis and clinically diagnosed patients **(a**, **b)** Overall and prostate cancer-specific survival comparison among PSA screened, PSA screened with later diagnosis and clinically diagnosed patients. **(c**, **d)** Overall and prostate cancer-specific survival comparison between PSA screened and clinical diagnosed patients. **(e**, **f)** Overall and prostate cancer-specific survival comparison between PSA screened patients and PSA screened with later diagnosis. **(g**, **h)** Overall and prostate cancer-specific survival comparison between PSA screened with later diagnosed and clinically diagnosed patients.

For overall survivals, five-year survivals were 87.66%, 69.19% and 74.46%, respectively, and 10-year survivals were 68.50%, 39.05% and 64.16%, respectively in PSA-screened, PSA-screened with later diagnosis and clinically diagnosed groups (Figure 4a, *P*<0.0001). There were also statistically significant differences between any of the two groups, including patients from PSA-screened versus clinically diagnosed (Figure 4c, *P*=0.0054), PSA-screened versus PSA-screened with later diagnosis (Figure 4e, *P*<0.0001) and PSA-screened with later diagnosis versus clinically diagnosed (Figure 4g, *P*=0.0199).

Prostate cancer-specific five-year survivals were 90.92%, 69.19% and 76.31%, respectively, and 10-year survivals were 82.72%, 41.74% and 65.76%, respectively in the PSA-screened, PSA-screened with later diagnosis, and clinically diagnosed groups (Figure 4b, *P*<0.0001). Statistically significant differences were found when comparing PSA-screened with clinically diagnosed (Figure 4d, *P*<0.0001), PSA-screened versus PSA-screened with later diagnosis (Figure 4f, *P*<0.0001) and PSA-screened with later diagnosis versus clinically diagnosed groups (Figure 4h, *P*<0.0278).

## DISCUSSION

To the best of our knowledge, this study done between 1996 and 2012 by the Center of Diagnosis, Treatment and Research of Prostate Disease of Jilin University and JICA is the first PSA-based mass screening project in China. Our study is also the first in China to compare the clinical and survival characteristics of prostate cancer patients diagnosed through PSA mass screening with those of clinically diagnosed patients.

Prostate cancer is one of the most common malignancies among males [1]. PSA-based mass screening has been widely used in the United States since the late 1980s. Although it caused a dramatic increase in the detection of prostate cancer in late 1980s and early 1990s, it is presumed to be responsible for the lowered mortality reported both in the United States and European countries [2, 3]. In the United States, the five-year survival was reported over 97% from 2006 to 2012 for all stages of prostate cancer [7] and an European randomized study showed a more than 90% prostate cancer-specific survival after a median follow-up of around 13 years [10]. However, in our study, the five-year and 10-year survival rates in a Chinese cohort were much lower, at 77.52% and 62.57%, respectively for overall survival and 79.65% and 68.60%, respectively, for prostate cancer-specific survival. This discrepancy possibly can be explained by decades-long routine usage of PSA-based mass screening in Western countries. Early-stage diagnosis of prostate cancer generally enabled many different treatment options, including curative management, to be possible [17, 22].

PSA level, T staging and Gleason score are still the three important diagnostic markers and outcome predictors [11]. As expected in our study, we found that men with metastases had a significantly high PSA level and significant differences in T staging and Gleason score. Prostate cancer can be stratified into different risk groups based on metastatic status and management approach and men free from metastases or treated by radical prostatectomy had better prognosis with significantly longer survival time. Moreover, as a highly heterogeneous disease with respect to survival time and disease recurrence, prostate cancer was subdivided into different risk groups for better management selection. The NCCN classification, as one of the stratification systems, categorizes prostate cancer into low, intermediate and high risk [11]. Our study showed that prostate cancer can be stratified into different risk groups based on the predictors Gleason score and T staging of the NCCN classification and T staging was further confirmed as a significant prognostic predictor by COX Proportional Hazard model. However, as a long-term retrospective study, the PSA, T staging and Gleason score information of a small portion of patients diagnosed in 1980s and early 1990s was incomplete or uncertain and may have had some influence on the survival comparison based on NCCN classification. Hence, more studies need to be done before the NCCN classification can be used for the life expectancy evaluation in prostatic adenocarcinoma patients in China.

In the past decade, PSA-based mass screening has become controversial owing to the uncertain balance between the benefits of screening and the harmful effects of overdiagnosis and overtreatment [12, 13]. In 2013, Ilic D et al. reviewed 5 randomized controlled trials (RCTs) with a total of more than 340,000 men who had had PSA-based screening and found no statistically significance in prostate cancer-specific mortality between the screened and control groups [14]. Of the five RCTs, 2 done by the European Randomized Study of Screening for Prostate Cancer (ERSPC) and the US Prostate, Lung, Colorectal and Ovarian (PLCO) Cancer Screening Trial were indicated to have a low risk of bias but presented contradictory results [14]. The former reported a significant reduction in prostate cancer-specific mortality attributable to screening at 9, 11 and 13 years of follow-up, while the latter showed no significance [4, 15–17]. In our study, PSA-based mass screening detected 358 cases of prostatic adenocarcinoma, including 259 immediate and 99 later diagnoses. Consistently, we found more early-stage prostate cancer in PSA-screened patients, with a significantly higher percentage in T1-2, N0 and Gleason≤6 subgroups, compared with patients who had had PSA screening with later diagnosis or a clinical diagnosis. The results resemble the situation in Western countries at a time when PSA-based mass screening was not used routinely [18, 19] which indicated that PSA-based mass screening was able to detect most still organ-confined and potentially curable prostate cancer patients and to improve survival. Our data are also supported by a report from the United States that following a 2012 USPSTF recommendation that discouraged routine PSA screening, there was a decrease in the detection of prostate cancer at an early stage. Another study concluded that the USPSTF recommendation was followed by an increase in metastatic prostate cancer [7, 10]. A retrospective multi-center study documented a high incidence of advanced prostate cancer in a Chinese cohort due to the absence of PSA-based mass screening [20]. More importantly, we found that overall and prostate cancer-specific survival rates were significantly higher in PSA-screened patients than in the PSA-screened patients with later diagnosis and the clinically diagnosed patients. Statistically significant differences were found when any two of the three groups were compared.

PSA-based mass screening has not been widely used in China yet but has been in routine use in the United States and European countries for decades. As a result, by the time a diagnosis of prostate cancer is made in China, the disease is much more advanced, resulting in a higher death rate [8, 9, 11]. Considering its small sample size and the short length of follow-up, our study may not fully reflect the effect of PSA-based mass screening on the Chinese cohort, so this study alone may not be sufficient to justify a recommendation for the routine use of PSA-based screening in China. However, based on the results of our study and the long-term trends and proven benefits of mass screening in the Western countries, we suggest that at least one nation-wide PSA-based mass screening at age>50 could be done in China to identify the potentially large number of asymptomatic patients and to reduce mortality caused by advanced prostate cancer and associated health care costs [21, 22]. In addition, considering the inequality of medical health resource allocation in China, there could be an opportunity for primary care physicians to gain awareness and training in PSA-based screening, especially in less developed areas.

In summary, we report here that a Chinese cohort has lower overall and prostate cancer survival rates than it is reported in western countries. The metastatic status, management approach, and T staging and Gleason score of the NCCN classification are significant predictors of both overall and prostate cancer-specific survivals. Most importantly, the incidence of early-stage prostate cancer found in PSA-based mass screening was high and PSA-screened patients had both a prolonged overall and prostate cancer-specific survival in comparison to clinically diagnosed patients. Our study suggests that at least one nation-wide PSA-based mass screening at age>50 could be done in China to identify the potentially large number of asymptomatic patients and to reduce mortality caused by advanced prostate cancer and associated health care costs.

## MATERIALS AND METHODS

### Study cohort

The study cohort consisted of 1,012 prostate cancer patients diagnosed between August, 1980 and April, 2012. Of these patients, 383 were detected by the PSA-based mass screening program done by the Center of Diagnosis, Treatment and Research of Prostate Disease of Jilin University between 1996 and 2012, with either immediate or later diagnosis. Other data used for this retrospective study were collected from prostate cancer patients diagnosed and treated in any of the three affiliated hospitals of Jilin University (The First Hospital of Jilin University, The Second Hospital of Jilin University and the China-Japan Union Hospital of Jilin University), Changchun (Changchun, China).

A total of 19,808 men aged over 50 years old from six major companies and institutions in the urban area of Changchun were interviewed and screened for prostate cancer based on total serum PSA level determination using the Elisa assay kit (CanAGDiagnostics, Gothenburg, Sweden). Men with PSA levels>4.0 ng/mL and those with obstructive symptoms (irrespective of PSA levels) were recommended for transrectal ultrasound-guided systematic sextant biopsies and subsequent pathological analysis. Among these patients, 259 men agreed to biopsies and were diagnosed as having prostatic adenocarcinoma immediately (hereafter referred to as PSA screened patients). However, another 99 men refused the biopsies for final diagnosis at the first time although their PSA levels>4.0 ng/mL and were diagnosed as prostatic adenocarcinoma in later years when their PSA level climbed further to a very high level (hereafter referred to as PSA screened patients with later diagnosis).

Basic patient characteristics of patients were evaluated and recorded by physicians: year of diagnosis, age, PSA, management approach, clinical staging (Tumor Node Metastasis, TNM), Gleason score at the time of diagnosis and survival time when applicable. Sextant biopsies were performed by pathologists with expertise in genitourinary tumors for Gleason score determination. Curative management was defined as radical prostatectomy only in this study. Metastatic prostate cancers were defined as the patients with distant or nodal metastasis at the time of diagnosis. Follow-up began after treatment and the cut-off date for death determination was 15 August, 2016. Survival time was defined as duration from the date of diagnosis to deceased date or census date.

Clinical characteristics of the study cohorts stratified by age or metastatic status at diagnosis were investigated. Then both overall and prostate cancer-specific survival was compared for prostatic adenocarcinoma of PSA screened and clinically diagnosed patients based on clinical characteristics and the National Comprehensive Cancer Network (NCCN) risk classification. Cox Proportional Hazards Model analysis was done for prostatic carcinoma patients for risk predictors identification. Furthermore, clinical characteristics and survival were further compared among PSA screened, PSA screened with later diagnosis and clinically diagnosed prostatic adenocarcinoma patients to evaluate the effect of PSA-based mass screening in China.

### Statistical analysis

Distribution of categorical clinical characteristics, including TNM staging, Gleason score, management approach and year of diagnosis, were compared using the Pearson Chi-Square test. Mann-Whitney test was used to compare PSA level, age at the time of diagnosis and follow-up time since the data were not in a standard normal distribution. The log-rank test was used to analyze the distribution of survival time. COX proportional hazard model was applied to identify significant predictors using a backward variable selection method with an elimination criterion of 0.05, and parameters included for analysis were age at diagnosis, year of diagnosis, PSA levels, Gleason score, clinical staging and management approach. Results were considered statistically significant when *P*<0.05.
